# The STEAM learning performance and sustainable inquiry behavior of college students in China

**DOI:** 10.3389/fpsyg.2022.975515

**Published:** 2022-10-20

**Authors:** Liying Nong, Chen Liao, Jian-Hong Ye, Changwu Wei, Chaiyu Zhao, Weiguaju Nong

**Affiliations:** ^1^School of Education and Music Hezhou University, Hezhou, China; ^2^College of Tourism and Sport Health, Hezhou University, Beijing, China; ^3^Faculty of Education, Beijing Normal University, Beijing, China; ^4^Kindergarten Training Center Ministry of Continuing Education, Guangxi College for Preschool Education, Nanning, China; ^5^School of Education, Guangxi University of Foreign Languages, Nanning, China

**Keywords:** Chinese teacher education student, collaborative self-efficacy, perceived usefulness of short videos, perceived ease of use of short videos, STEAM learning performance, sustainable inquiry behaviors

## Abstract

Teacher education students, as an important reserve in the field of education, their growth and development are related to the future of science, economy, sustainable development of education. Through participation in the educational practice of STEAM, which integrates science, technology, engineering, art, and mathematics is more beneficial for them to acquire 21st century skills like communication, collaboration, learning innovation and critical thinking. However, little has been seen about the use and effectiveness of short videos in STEAM education activities and how they can be used to support and promote teacher education students STEAM learning performance and sustainable inquiry behaviors. Therefore, this study combines the framework of triadic reciprocity determinism theory and technology acceptance theory to propose six research hypotheses and develop a research model to explore the relationship between collaborative self-efficacy (CS-E), perceived usefulness (PU) of short videos, perceived ease of use (PEOU), STEAM learning performance and teacher education students’ sustainable inquiry behavior. To achieve the purpose of this study, this study used purposive sampling and invited teacher education students from five classes of preschool education at a university in Guangxi Zhuang Autonomous Region (GZAR), China, to participate in this study. A total of 8 h of instructional guidance was provided over a four-week period, in which participants used short videos for collaborative group learning and independent inquiry and applied STEAM concepts to kindergarten science activities. Participants’ STEAM performance was measured and surveyed during the fourth week. The results of the study validation showed that (1) teacher education students CS-E had a positive effect on the PU and PEOU of short videos; (2) Teacher’s education students PEOU of short videos had a positive effect on usefulness; (3) Teacher’s education students PU and PEOU of short videos had a positive effect on STEAM learning performance; (4) Teacher’s education students’ STEAM learning performance had a positive effect on sustainable inquiry behavior.

## Introduction

According to Sustainable Development Goal 4 (SDG4), an initiative of the United Nations (UN) for 2030, achieving quality education is an important issue for achieving sustainable development in education (UN, 2020). Especially with the rapid development of technology, economy and society, the demand for science, technology, engineering, and education personnel is increasing. Therefore, collaborative inquiry learning, critical thinking, and problem-solving skills have become important elements affecting the sustainability of education (Lu et al., 2022). Among the many approaches to education, STEAM is a multidisciplinary approach involving science, technology, engineering, arts, and mathematics (Shen et al., 2021), which emphasizes the importance of continuous inquiry, collaboration, learning, and growth in hands-on activities to help students better prepare for lifelong learning (Rahmawati et al., 2022). STEAM programs, on the other hand, provide more learning opportunities and continue to promote students to become better lifelong learners through collaboration, inquiry, and learning in a variety of formal and informal contexts (Domenici, 2022).

As STEAM programs expand globally, they are not only changing the curriculum paradigm in primary and secondary schools around the world, but are also becoming an important frontier for curriculum reform in higher education (Selcen Guzey et al., 2017), as well as making STEAM a highly valued educational approach (Sha et al., 2021). Among them, the growth and development of teacher trainees, as an important reserve in the field of education, are related to the future sustainability of science, economy, and education (Nousheen et al., 2020), and through participation in educational practices that integrate STEAM, it is more beneficial for them to acquire 21st century skills such as collaboration, digital learning, and critical thinking (Partnership for 21st Century Skills, 2019; Singh, 2021). In addition, STEAM programs are crucial for teacher trainees because through hands-on STEAM activities they can not only enhance their motivation, creative thinking, and problem-solving skills (Ültay and Ültay, 2020), but also improve the core literacy of future teachers and better facilitate students’ independent learning, collaborative inquiry, and development in future educational teaching.

In addition, STEAM programs focus on interdisciplinary and inquiry-based learning, and engaging in more STEAM activities will help improve student performance and continue to influence their effectiveness in inquiry activities (Cheng et al., 2021). However, many studies have found that participation in STEAM is not optimistic, as evidenced by low awareness and interest in science and engineering, low awareness of active learning in STEAM programs, insufficient STEAM program resources, and even program overload (Jesionkowska et al., 2020). Such a situation, if perpetuated, will not only affect teacher education students’ STEAM performance, but also their continued inquiry behavior. Exploring teacher education students’ STEAM learning performance and sustainable inquiry behaviors is therefore critical to how to achieve educational sustainability.

In addition, collaborative efficacy, a group’s confidence, and belief in working together to achieve organizational goals (Bandura, 1977), affects people’s engagement and persistence in group activities, and is an important predictor of student motivation, performance, and outcomes (Fan and Ye, 2020; Hong et al. 2020a; Talsma et al., 2021). More importantly, collaborative learning in the STEAM learning process is believed to be related to students’ cognition, motivation to learn, and to work and explore with their peers to influence their learning outcomes (Nguyen et al., 2021). The learning environment of teacher trainees is often filled with critical events and challenges that require continuous preparation for becoming future teachers through self-directed learning, participation in practice, and enhancement of competencies. Collaborative learning can not only stimulate their self-efficacy, but also further prevent students from academic failure through self-directed learning (Fernandez-Rio et al., 2017). Therefore, in STEAM inquiry activities, teacher education students’ collaborative self-efficacy (CS-E) is critical and a key factor influencing teacher education students’ learning performance and sustainable inquiry behavior.

In addition, social media is a good medium for science education, and the widespread use of social media such as short videos around the world not only breaks the barriers of time and space to attract people to view videos (Choi et al., 2021), but also continues to expand learners’ information and vision with its diverse content, vivid images, and interactive experiences (Mustafa et al., 2021). In China, with the continued growth of short video platform applications such as Douyin (TikTok), Kuaishou, Huoshanzhibo, and Little Red Book, the number of short video users in China was expected to reach 870 million by 2021 (China Internet Network Information Center, 2021). Such platforms are popular for instantly creating and sharing moments of life and work.

More importantly, with the rapid development of educational App in education (Papadakis et al., 2020), they not only provide more communication platforms and messages for parents and students (Vaiopoulou et al., 2021), but also facilitate students’ active participation in various informal and formal learning environments (Vaiopoulou et al., 2022). Studies have shown that short videos can promote pre-service students’ self-efficacy by sharing more video resources, which also makes them more likely to use short videos to complete learning tasks (Liu et al., 2021). Teacher trainees, as a reserve of future teachers, should use information technology to facilitate collaborative learning and development of students (Masruddin, 2018). Therefore, through the learning and application of short videos, we can not only increase teacher trainees’ learning engagement, but also promote their collaborative learning and inquiry behaviors (Zhao et al., 2021). Thus, short videos play an important role in teacher education students’ learning performance and sustainable inquiry behavior.

According to the triadic reciprocity theory proposed by Bandura (1986), human behavior is the result of the intertwined influence and continuous action of the individual and the environment, where the environment, the individual, and the behavior are interconnected and continue to act on the individual’s behavior and outcomes in a reciprocal and dynamic manner. In the current highly changing social media environment, people’s attitudes toward short videos and the effects of their use will influence their behavior and outcomes (Pan et al., 2022). Therefore, the triadic reciprocity theory can be used to explain learners’ continuous enhancement of individual cognition under the influence of social media environments, which in turn affect learners’ behavior and performance in a complex interactive manner (Hu et al., 2016). Therefore, using the triadic reciprocity theory as a theoretical basis for teacher education students’ learning performance and continuous inquiry behavior will help strengthen the study.

In addition, the Technology Acceptance Model (TAM) theory can be used to explain the process of technology acceptance from external to internal variables, simulating the behavioral analysis process of individuals’ acceptance and use of technology in a social media environment (Sánchez-Prieto et al., 2017). In the STEAM learning process, the perceived usefulness (PU) and perceived ease of use (PEOU) of STEAM education not only affects learners’ attitudes toward learning, but also continues to influence their inquiry and learning outcomes through STEAM course learning (Huang and Liu, 2021). Also, related studies have shown that social media play an important role in increasing learners’ willingness to learn and cooperate in learning activities (Sánchez-Prieto et al., 2017).

However, in the past research, little has been seen about the use and effectiveness of short videos in STEAM education activities and how they can be used to support and promote teacher education students’ STEAM learning performance and sustainable inquiry behaviors. In addition, according to Sustainable Development Goal 4 (SDG4) 2030, collaborative inquiry learning is an important issue for achieving sustainable development in education. Therefore, based on SDG4, this study combines the framework of triadic reciprocity theory and technology acceptance theory to develop a research model that explores the relationship between CS-E, PU of short videos, PEOU, STEAM learning performance, and teacher education students’ sustainable inquiry behaviors. Based on the above research objectives, the research question is therefore proposed as follows:

Q1. Does teacher education student’s CS-E affect their PU and PEOU of short videos?

Q2. Does the perceived PEOU of short videos by teacher education students affect the PU of short videos?

Q3. Does the PU and PEOU of short videos by teacher education teachers affect their STEAM learning performance?

Q4. Does teacher education teachers STEAM learning performance affect sustainable inquiry behavior?

## Research hypothesis and model

### Theoretical framework and research model

Bandura's (1986) triadic reciprocity theory suggests that human behavior is the result of interaction between the individual and the environment, and that the environment, the individual, and the behavior are dynamically and reciprocally interconnected and continue to act on the individual’s behavior and outcomes. In addition, social media environments such as short videos can influence learners’ performance and behavior by enhancing their cognition with better interactions (Hu et al., 2016). Therefore, in this study, based on Bandura’s reciprocal determinism, the “environment” is considered as the social media environment such as short videos, the “individual” is considered as the teacher education students who uses short videos to participate in STEAM learning activities, and the “behavior” is considered as the teacher education students’ CS-E, STEAM learning performance, and continuous inquiry behavior. That is, in the current social media environment such as short videos, higher PU, and PEOU of short videos by teacher education students may be beneficial for stimulating their CS-E in STEAM, which will continue to influence their learning performance and inquiry behaviors.

**Figure 1:**
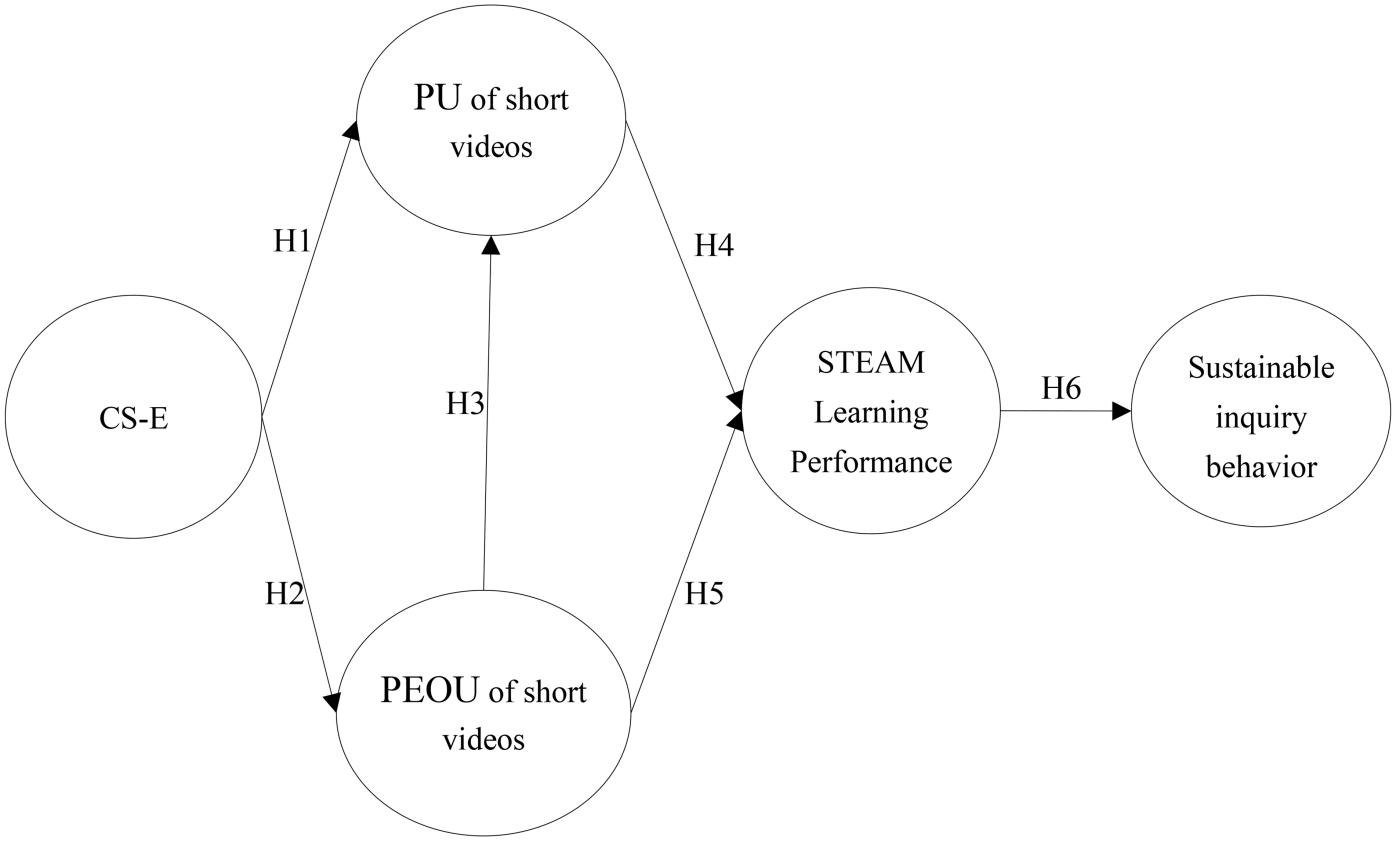
Research model.

In addition, the Technology Acceptance Model (TAM) proposes that the key factors of system technology acceptance consist of external variables, PEOU, PU, attitudes toward use, and behavioral intentions that together assess the technology acceptance of users in a system (Davis, 1989; Chatzopoulos et al., 2022). According to the TAM model theory, people’s technology acceptance can be understood from external variables to internal variables, with PU being how much individuals value the technology they use, and PEOU being how easy individuals perceive the technology to be to operate (Davis, 1989). Therefore, in today’s social environment with the rapid development of social media such as short videos, applying external variables such as short videos to STEAM course learning is more conducive to increasing learners’ internal motivation to influence their inquiry behaviors and outcomes through STEAM course learning with short video applications (Huang and Liu, 2021). Therefore, this study combined triadic reciprocity theory and TAM theory to examine the relationship between teacher education students’ CS-E, PU of short videos, PEOU, learning performance, sustainable inquiry behavior, and constructed the following research model architecture diagram (see Figure 1).

### Research hypothesis

#### The effect of CS-E on PU and PEOU of short videos

According to social cognitive theory, self-efficacy is an individual’s belief and confidence in accomplishing a task (Bandura, 1977) and provides learners with sustainable energy that influences learning behavior and outcomes (Caprara et al., 2008). Learners who have higher collaborative efficacy tend to have stronger collaborative beliefs and better collaborative behaviors (Hong J. C. et al., 2021). Related studies also indicate that teamwork self-efficacy is important because it affects not only students’ collaborative learning but also their attitudes toward using information technology (Konak et al., 2019). In addition, students’ self-efficacy is related to the PU and PEOU of technology (Pan, 2020), through which learners can feel that technology is more useful and easier to use (Nur and Joviando, 2021). As an important form of social media, short videos are more visible, more interactive, and richer resources to facilitate students’ learning (Mustafa et al., 2021). Since teacher education students’ need more collaborative learning to achieve common learning goals in the learning process, if they can learn through the application of short videos in the collaborative learning process, it will not only improve their CS-E, but also be more beneficial for them to complete the corresponding collaborative learning and inquiry tasks (Zheng et al., 2020). Therefore, based on the aforementioned literature, this study proposes the following hypotheses regarding teacher education students’ CS-E and PU and PEOU of short videos:

*H1*: CS-E has a positive effect on PU of short videos.

*H2*: CS-E has a positive effect on PEOU of short videos.

#### The effect of PEOU of short videos on PU of short videos

TAM theory is mainly used to explain people’s technology acceptance (Davis, 1989), and PEOU and PU are the core components of TAM theory, which are interrelated and used to predict people’s behavior and attitudes toward technology perception in various research areas (Choi et al., 2021). Studies have found that video platforms such as YouTube make it easy for learners to complete learning tasks by watching short videos in informal learning environments by providing more video resources (Hong et al., 2020a). In addition, as ICT technologies continue to develop, short videos will also be visual and interactive, which will be more useful for teacher education students to apply in their collaborative learning (Moreno-Guerrero et al., 2020). More importantly, short videos may be important for teacher education students’ motivation and performance because they can use them to learn more about learning, and as a learning resource for ongoing learning and inquiry in a variety of formal and informal learning settings (Zhao et al., 2021). Therefore, the hypothesis of PEOU of short videos on PU of teacher education students’ is proposed in this study as follows:

*H3*: PEOU of short videos has a positive effect on PU of short videos.

#### The impact of PU and PEOU of short videos on STEAM learning performance

Research has found that information technology (ICT) is critical for students because not only does it change the way they learn, but it also has a lasting impact on students’ learning behaviors and performance (Garcia-Martinez et al., 2021). Furthermore, according to the TAM model theory, the PU and PEOU of perceptual technology may play an important role in students’ STEAM learning outcomes (Davis, 1989; Huang and Liu, 2021). Studies have also found that teachers who perceive IT to be important are more likely to perceive the benefits and PU of IT in their educational work, and to further increase their behavioral intentions to continue using it through the perceived convenience and operability of the technology (Hong X. M. et al., 2021. Therefore, with the use of social media such as short videos in various fields, there is a surge of interest in social service platforms such as short videos, and they are considered as an important resource for educational teaching (Slemmons et al., 2018). Studies have also shown that more short video resources used in the learning process can help teacher education students improve their self-efficacy and performance (Zhao et al., 2021). More importantly, STEAM activities focus more on the variety of formal and informal contexts in which inquiry learning can take place, and short videos are more conducive to helping students expand their learning resources. Therefore, if teacher education students can perceive the importance and value of short videos and can feel the PEOU of short videos, it will help to improve their STEAM learning performance. Therefore, this study proposes the following research hypotheses:

*H4*: PU of short videos has a positive effect on STEAM learning performance.

*H5*: PEOU of short videos has a positive effect on STEAM learning performance.

#### Impact of STEAM learning performance on sustainable inquiry behavior

As sustainable development (SD) issues continue to have an impact in various organizational areas of people’s lives, they are also accompanied by changes in people’s behavior patterns (Podgórska and Zdonek, 2022). Related studies have shown that information technology not only improves students’ learning performance and motivation, but also continuously influences students’ active learning behaviors (Hwang et al., 2018). Also, a study by Wen et al. (2020) found that students with better learning performance in a guided inquiry learning environment were more actively engaged in inquiry activities and demonstrated good scientific literacy. STEAM education, on the other hand, is a multidisciplinary approach that emphasizes the development of higher-order thinking and competencies (Shen et al., 2021), with more emphasis on hands-on activities that promote the development of sustainable inquiry behaviors (Rahmawati et al., 2022). In addition, in the process of educational sustainability, teacher education students need to promote the development of their collaborative, creative, and thinking skills through STEAM learning (Ültay and Ültay, 2020), and to improve their learning performance through course work that better promotes continuous inquiry behaviors. That is, students with higher academic achievement tend to have higher inquiry behaviors and, conversely, students with lower academic achievement tend to have lower inquiry behaviors (Chen and Wang, 2020). Therefore, learning performance is critical for teacher education students’ sustainable inquiry behavior. Therefore, this study proposes the following hypothesis based on the above literature:

*H6*: STEAM learning performance has a positive impact on sustainable inquiry behavior.

## Research design

### Course design

Science education for preschool children is one of the required professional foundation courses for preschool education teacher-training students in Hezhou University, China, and the use of STEAM education methods in this course is more conducive to improving students’ collaborative inquiry learning ability. In the science education program for preschool children, students are introduced to STEAM knowledge in preschool education through STEAM science, STEAM technology, and STEAM art. Therefore, to promote teacher education students’ participation in STEAM collaborative learning and inquiry activities, this study arranged a 4-week quasi-experimental design with 2 h per week for STEAM knowledge learning and application to understand STEAM knowledge and its application in preschool education.

**Figure 2:**
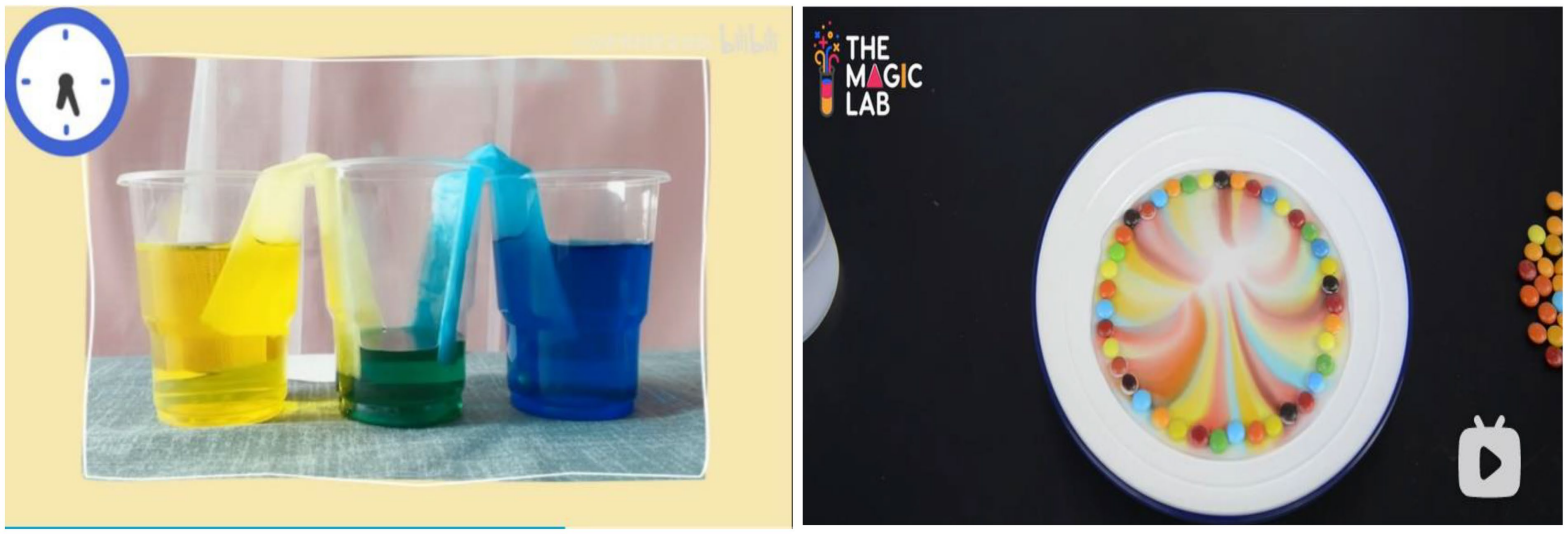
Short video screenshot.

**Figure 3:**
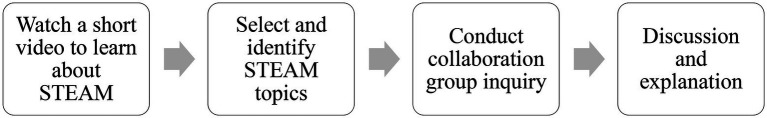
Course content.

Before each STEAM course, teacher trainees need to use short videos to carry out independent collaborative group learning to learn about kindergarten STEAM education activities in the field of preschool education, and in addition, they must use short video resources to design kindergarten STEAM education activities in the process of carrying out collaborative group learning. In addition, in the weekly 2-h course, students are first guided through STEAM concepts in combination with short videos (Xiaomin Sunshine, 2020; Children’s Scientific Enlightenment, 2022), which can see in Figure 2. Such as, such as the properties and applications of various materials related to science, the use of tools and ways of investigation related to technology, structural design and effects related to engineering, aesthetic experience and creative practice related to art, and scale measurement and quantitative relationships related to mathematics. Secondly, students are guided to observe, predict, investigate, manipulate, discuss, and explain in a collaborative group setting to understand the application of STEAM in preschool education, such as “Paper Boat Power” and “Play with Sushi.” The teacher then gives timely feedback based on their performance and discusses ways to enhance their STEAM activities. Finally, the teacher will confirm and guide the students as they revise and continue to explore with the video resources, as shown in Figure 3.

### Research methodology and procedures

Quasi-experimental designs allow for better evaluation of experimental effects and can avoid the randomness and uncontrollability associated with teaching practices (Kim and Steiner, 2016). Purposive sampling, a more common sampling method for validation studies, would facilitate a better match between the study sample and the study purpose (Vogt, 2007). Therefore, in this study, a total of five classes of teacher-training students in preschool education at Hezhou University, China, were invited to participate in this teaching experiment using a stance sampling approach. In order to explore how these teacher-training students use short videos to carry out collaborative inquiry learning of STEAM knowledge in their preschool children’s science education course learning, in the 4-week teaching experiment, firstly, the teacher-in-charge introduced the experiment process, secondly, the preschool teacher trainees were guided to learn the core concept of STEAM and how to apply it in the field of preschool education, and then they were guided to learn about the application of STEAM in the field of preschool education by searching for short videos on the Internet through the weekly STEAM group collaborative learning tasks, and to conduct independent inquiry learning through group collaboration. In addition, the Wènjuànxīng (WJX) platform is an online survey platform similar to Google Form, which allows students to fill out questionnaires online in an anonymous way. Therefore, during the last week of the course, students were guided to fill out surveys and STEAM learning performance quizzes through the Wènjuànxīng (WJX) platform.

### Participants

In this teaching experiment, 250 sophomore and junior teacher-training students majoring in preschool education in Guangxi Zhuang Autonomous Region, China, were invited to participate in this study using a purposive sampling method. There were eight invalid questionnaires due to incomplete answers, short response time, and extreme values, so the number of valid participants in this study was 242, with a valid return rate of 96.8%. Of these, six (2.5%) were males and 236 (97.5%) were females. There were 140 sophomores (57.9%) and 102 juniors (42.1%). There were 76 vocational students (31.4%), 33 (13.6%) in high school, 83 (34.3%) in secondary school, and 50 (20.7%) in college; 20 of the respondents (8.3%) watch short videos 1–2 days a week, 27 (11.1%) watch short videos 3–4 days a week, 23 (9.5%) watch short videos 5–6 days a week, and 172 (71.1%) watch short videos every day. The most popular short video platforms are TikTok with 137 respondents (56.6%), Little Red Book with 54 (22.3%), Kuaishou with 33 (13.7%), and Huoshanzhibo with 18 (7.4%).

### Measurements

To achieve the purpose of this study, it combined previous research and related theories to develop a corresponding measurement tool, and invited three experts in the field of education to review the measurement tool, first to review the rationality and completeness of the theme and question items of the measurement tool, then to make corresponding suggestions based on the readability of the revised theme, and then to review and suggest the fluency of the revised theme. After conducting the expert review, five preschool teacher education students were invited to try out the measurement instrument and fill out the responses. Therefore, to increase the reliability of the study, the study was rated using a Likert 5-point scale, where 1–5 indicates *strongly disagree* to *strongly agree*.

#### Collaborative self-efficacy

Self-efficacy, as an assessment of an individual’s cognitive and belief states, usually refers to an individual’s confidence and beliefs about his or her ability to accomplish a specific task or goal (Bandura, 1986), so this study adapted (Hong et al.’s, 2020b) Collaborative Efficacy Questionnaire with a total of eight questions to assess teacher education students’ CS-E based on the above definition. Examples: “In working with the group on the assignment, I believe that our group can work as a team to complete the assignment” and “In working with the group on the assignment, I believe that our group can make full use of each student’s ability to achieve the goal.”

#### PU of short videos

PU refers to the degree to which individuals value the technology they use in the process of using it (Davis, 1989), and according to this definition, this study adapted Hong et al.’s (2011) PU of Technology Scale with six questions to assess the PU of short videos for teacher education students. Example items are: “I think short videos can inspire my interest in STEAM learning” and “Using short videos, I can keep learning about STEAM.”

#### PEOU of short videos

PEOU is the ease with which an individual perceives a technology to operate in the process of using it (Davis, 1989). In order to understand the PEOU of short videos by teacher education students, this study adapted (Hong et al.’s, 2011) PEOU of technology scale with six questions based on the above definition. The content of the questions is as follows: “It is easy for me to browse short videos on short video apps to learn” versus “It is easy for me to use the features of short video sites to watch certain short videos over and over again to learn.”

#### STEAM learning performance

To understand teacher education students’ STEAM learning performance, this study designed quizzes from five modules in the field of preschool education on science, technology, engineering, art, and mathematics, with four questions in each module. Sample questions in the science field are as follows: “Which material is most likely to become a paste when paper is stirred and dissolved in water?” and “What materials can be used to explore the materials involved in the science activity ‘Making Fluffy 3D Paintings’?” Examples of the technology area are: “What tools can you use to make delicious cheese?” and “What methods do you need to use when doing the middle school science activity ‘Paper Bridge is Powerful’?” Examples in the field of engineering include “Which ingredient mixes with glutinous rice flour and water to create the most viscosity?” and “How can you use building blocks to form a robot and adjust it to optimize it?” Examples in the art field include “Which group of colors makes the most coherent pinball table?” and “How can you make the windmill rotate in a way that creates a noticeable color change?” Examples in the area of mathematics include: “What is the mathematical knowledge involved in the middle school science activity ‘Paper Bridge is Powerful’?” and “Which of the following sizes of paper flowers bloom the fastest on the water surface?”

#### Sustainable inquiry behavior

Inquiry is a way for learners to ask questions, seek information, and solve problems (Brondfield et al., 2019). Through the act of continuous inquiry, learners can autonomously enhance their knowledge and influence their learning outcomes (Schmid and Bogner, 2015). Therefore, based on the above definitions, this study developed its own sustainable inquiry behavior questionnaire with six questions to assess the continuous inquiry behavior of teacher education students. For example, “I will continue to use inquiry-based approaches to learn in the future” and “I will want to continue to participate in inquiry-based courses in the future.”

## Results and discussion

In the social sciences, structural equation modeling (SEM) is to explain and test structural relationships among research variables and hypothesized associations among indicators and can better explain the relationships among potential variables by modeling the measured variables (Deng et al., 2018). Based on this, to test the research model, this study used SPSS 25.0 for demographic variable analysis, correlation analysis, and reliability analysis, followed by AMOS 24.0 for item analysis, and SEM for model fitness testing and path analysis of the study model.

### Item analysis

First-order validation factor analysis can help researchers better identify items that do not meet the criteria and test the applicability of the original items (Kline, 2015). According to Kenny et al. (2015), the factor loadings (FL) should be higher than 0.500, so this study first analyzed the factor loadings of each item and removed the question items with FL lower than the value of 0.500. Secondly, to test the internal validity of the items and to have a good fit, the χ^2^/*df* suggested that less than 5, RMSEA less than 0.1, and GFI greater than 0.80 (Hair et al., 2019), and all four constructs of this study met the above standards (see Table 1). As a result, CS-E was reduced from eight to six questions; PU from six to four questions; PEOU from six to four questions; and sustainable inquiry behavior from six to five questions.

**Table 1:** First-order CFA.

Construct	χ^2^	df	*χ*^2^/df	RMSEA	GFI	AGFI	FL
Threshold	–	–	<5	<0.10	>0.80	>0.80	>0.5
CS-E	17.2	9	1.91	0.61	0.98	0.94	0.66 ~ 0.81
PU	3.34	2	1.67	0.60	0.99	0.97	0.69 ~ 0.76
PEOU	3.37	2	1.68	0.53	0.99	0.97	0.64 ~ 0.77
Sustainable inquiry behavior	8.09	5	1.62	0.51	0.99	0.96	0.69 ~ 0.74

### Reliability and validity analysis

In terms of reliability, according to Cook and Beckman (2006), a Cronbach’s α values and a composite reliability value of 0.7 and above indicate that the statistics are internally consistent. In contrast, the Cronbach’s α values in this study ranged from 0.84 to 0.91 which meet the recommended standards, and CR values ranged between 0.81 and 0.87, both above 0.7 (Cook and Beckman, 2006).

In terms of validity, Factor Loadings (FL) and Average Variance Extracted (AVE) are common to measure the convergent validity of the item which had convergent validity (Hair et al., 2012). The FL value for the research construct should be gathering than 0.5, while the FL values for the four items in this study ranged between 0.71 and 0.73 which meet the recommended standards (Hair et al., 2019). In addition, if the AVE value is higher than 0.5, it means that the items of the research have good convergence validity of the reformulation surface and can be judged to be valid (Hair et al., 2011), while the four items in this study had a range of constructs from 0.51 to 0.54, all of which were higher than 0.50 (see Table 2).

**Table 2:** Reliability and validity analysis.

Construct	*M*	*SD*	*α*	FL	CR	AVE	*t*
CS-E	3.59	0.56	0.91	0. 73	0.87	0.54	9.80 ~ 12.05
PU	3.66	0.58	0.88	0. 73	0.82	0.53	9.31 ~ 9.99
PEOU	3.45	0.68	0.84	0.71	0.81	0.51	8.54 ~ 8.89
Sustainable inquiry behavior	3.65	0.55	0.88	0. 72	0.84	0.51	9.55 ~ 10.12

In addition, Awang (2015) suggested that the square root of AVR should be greater than the Pearson correlation coefficient of the remaining constructs to indicate good discriminant validity; all of the constructs in this study had discriminant validity (see Table 3).

**Table 3:** Discrimination validity analysis.

Construct	1	2	3	4	5
CS-E	(0.73)				
PU	0.64	(0.73)			
PEOU	0.62	0.68	(0.71)		
Learning performance	0.57	0.58	0.64	(1)	
Sustainable inquiry behavior	0.6	0.65	0.55	0.55	(0.71)

### STEAM learning performance analysis

According to the statistical results, the mean STEAM learning performance of the study participants was 11.13, with a standard deviation of 2.42, a median of 11, a minimum of 4, and a maximum of 17. The specific data can be found in Table 4.

**Table 4:** STEAM learning performance analysis (*N* = 242).

Construct	*M*	*SD*	*Med.*	*Mini.*	*Max.*
STEAM learning performance	11.13	2.42	11	4	17

### Model fit analysis

Structural equation modeling (SEM) can better confirm the differential acceptance of the data obtained from this research model (Stanley and Edwards, 2016). The value of χ^2^/*df* should be less than 5; RMSEA should be less than 0.10; GFI, AGFI, NFI, NNFI, CFI, IFI, and RFI should be higher than 0.800 (Abedi et al., 2015), while PNFI and PGFI could be not less than 0.50 (Hair et al., 2010). Therefore, the data of this study are as follows, χ^2^/*df* = 1.65, RMSEA =0.05, GFI = 0.91, AGFI = 0.88, NFI = 0.89, NNFI = 0.95, CFI = 0.97, IFI = 0.95, RFI = 0.87, PNFI = 0.77, and PGFI = 0.71, all of which indicated that the model fit was good.

### Path analysis

This study proposes six research hypotheses based on Triadic Reciprocity Theory and TAM theory, which conducted a research model to test them. In Figure 2, the results showed that CS-E had a positive effect on PU of short videos (*β* = 0.44***; *t* = 4.52) and PEOU of short videos (*β* = 0.71***; *t* = 8.21); PEOU of short videos had a positive effect on PU of short videos (*β* = 0.46***; *t* = 4.55); PU of short videos (*β* = 0.29**; *t* = 2.76) and PEOU of short videos (*β* = 0.47***; *t* = 4.37) had a positive effect on STEAM learning performance; and STEAM learning performance had a positive effect on sustainable inquiry behavior (*β* = 0.62***; *t* = 8.87).

**Figure 4:**
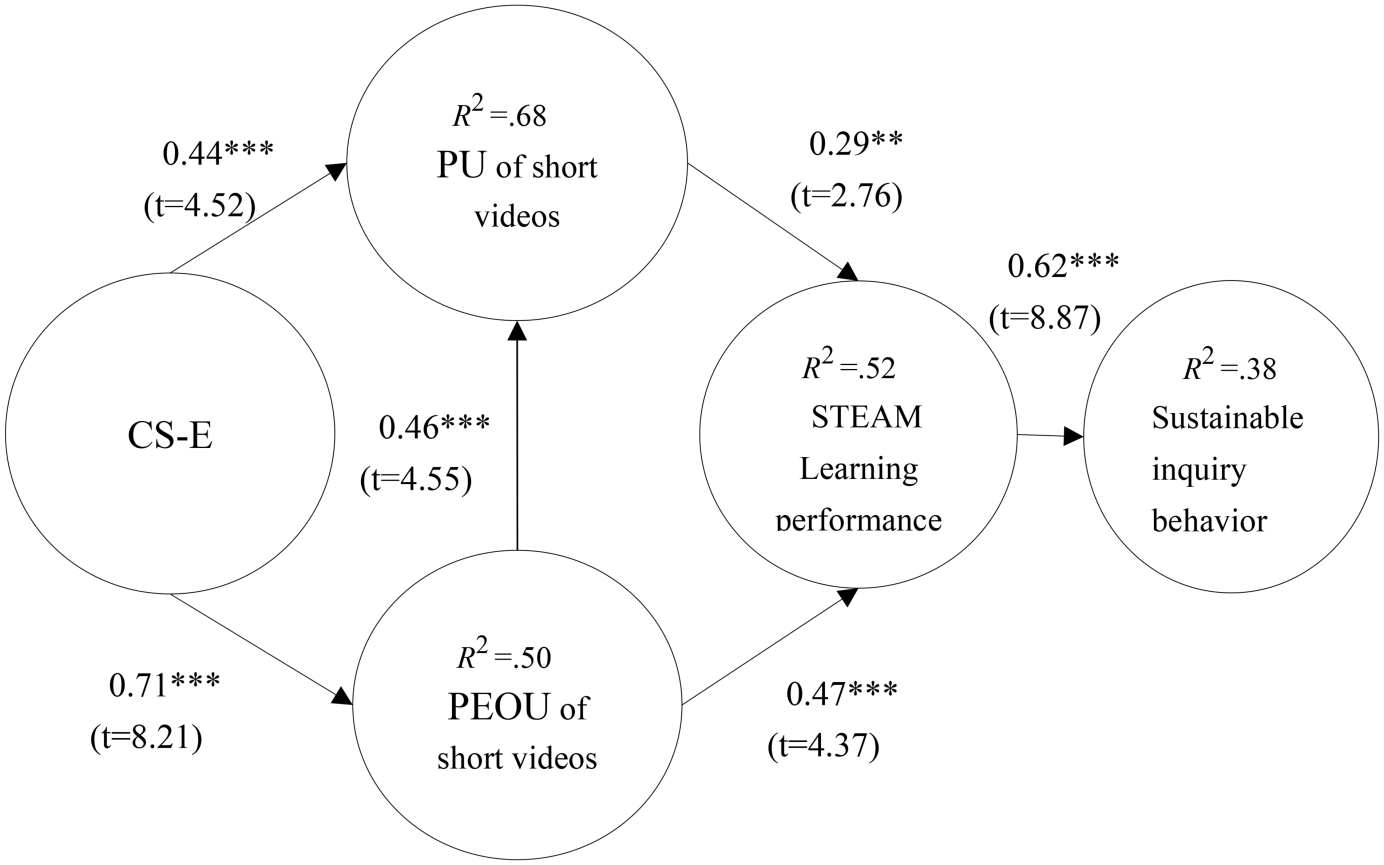
Validation of the research model. **p < 0.01, ***p < 0.001.

In this study, the explanatory power of PU of short videos was 68%, the explanatory power of PEOU of short videos was 50%, the explanatory power of STEAM learning performance was 52%, and the explanatory power of sustainable inquiry behavior was 38%. This indicates that this study has medium and strong or higher degrees of explanatory power (Hair et al., 2010), as shown as in Figure 4.

### Discussion

Achieving quality education and providing lifelong learning opportunities for all is an important element of SDG4 by 2030 (UN, 2020), with more focus on how to promote learner inquiry based on lifelong learning and the ability to innovate, among other things (Lu et al., 2022). Among them, collaborative efficacy, as an important motivational factor for learners, is a group confidence and belief in achieving a common goal and promotes people’s learning status through participation in group activities (Bandura, 1977; Hong et al., 2020b), and in this study, teacher education students showed positive attitudes towards CS-E (*M* = 3.59, *SD* = 0.56). In addition, PU is how much individuals value the technology used during technology acceptance, and PEOU is how easy individuals perceive it is to use and operate the technology (Davis, 1989; Huang and Liu, 2021). In this study, teacher education students showed a positive willingness to use short videos due to their PU (*M* = 3.66, *SD* = 0.58) and PEOU (*M* = 3.45, *SD* = 0.68). In addition, learning performance refers to the effectiveness of the application of the knowledge and competencies that learners have in the learning process (Pham and Huynh, 2018), and in STEAM course learning, teacher education students’ STEAM learning performance is not high (*M* = 11.13, *SD* = 2.42). Furthermore, inquiry refers to a way in which learners can influence their learning outcomes through asking questions, seeking information, and solving problems (Brondfield et al., 2019), and learners can influence their learning outcomes through independent learning and inquiry (Schmid and Bogner, 2015); however, in this study, teacher education students’ sustainable inquiry behaviors (*M* = 3.65, *SD* = 0.55) showed positive attitudes. The results of the study show that CS-E had a positive effect on both PU and PEOU of short videos, so H1 and H2 were positively verified. Perceived short-video PEOU had a positive effect on PU of short-video, so H3 was positively verified. PEOU and PU of short videos had a positive impact on STEAM learning performance, so H4 and H5 were positively validated. STEAM learning performance had a positive effect on sustainable inquiry behavior, so H6 was positively validated.

#### CS-E has a positive effect on PU and PEOU of short videos

According to Gully et al. (2002), teamwork effectiveness is often viewed as an assessment of an individual’s ability and confidence to participate in group activities. Generally, CS-E is strongly associated with good learning outcomes, as Hong et al. (2020b) found that when students have high collaborative efficacy, it will help them strengthen their collaborative beliefs as well as promote better collaborative behaviors. In addition, students’ collaborative attitude and self-efficacy during collaborative learning can enhance their interest in using IT (Konak et al., 2019) and they will be more willing to use IT in collaborative and inquiry learning. In addition, with the rapid development of social media such as short videos, those teacher education students who have more teamwork were found in Masruddin’s (2018) study to explore through group collaboration and were more likely to adopt short videos to enhance their learning. Conversely, without a good sense of CS-E, they will not be motivated to learn, and it will be detrimental to the effectiveness of accepting and using short video learning with collaborative learning (Zheng et al., 2020). The results of this study showed that teacher education students’ CS-E had a positive effect on PU and PEOU of short videos, which was consistent with previous studies. That is, the higher CS-E teacher trainees have, the better will be their PU and PEOU of short videos.

#### PEOU of short videos has a positive effect on PU

Davis (1989) proposed that technology acceptance is a process of change from external to internal variables based on TAM theory. In addition, several studies have confirmed that PEOU of technology is an important predictor of PU and is interlinked to influence individual technology acceptance (Choi et al., 2021; Hong X. M. et al., 2021). For teacher education students, social media such as short videos are more resourceful, interactive, and timely (Mustafa et al., 2021), making it easier for them to access appropriate learning resources from short video platforms, and they are also more willing to use short video resources in collaborative learning (Moreno-Guerrero et al., 2020), thus laying the foundation for being lifelong learners in their future work. They are therefore better prepared to become lifelong learners in their future educational endeavors. In addition, Zhao et al.'s (2021) study found that social media such as short videos are an important learning medium for teacher education students because they enable support for their continuous learning and development in a variety of formal and informal learning environments. Moreover, the results of this study showed that teacher education students’ PEOU of short videos had a positive effect on PU of short videos, which is consistent with previous studies. That is, the higher the PEOU of short videos by teacher education students, the higher their PU of short videos.

#### PU and PEOU of short videos have a positive effect on STEAM learning performance

Studies have found that, as important workers in the future field of education, teacher education students’ ICT application skills not only affect their ability to become better lifelong learners, but also have a significant impact on their learning performance (Zhao et al., 2021). In addition, based on the TAM model, Huang and Liu (2021) suggested that PU and PEOU of technology are important to students because they can enhance their learning performance in STEAM activities through the use of technology. In contrast, in this study, teacher education students’ PU and PEOU of short videos were significant predictors of STEAM learning performance, implying that PU and PEOU of short videos positively influenced teacher education students’ learning performance, which was consistent with prior research. That is, teacher education students who find short videos easy to manipulate tend to be more likely to adopt them as an important resource for science learning and to have better learning performance in STEAM learning activities.

#### STEAM learning performance has a positive effect on sustainable inquiry behavior

In SDG4, there is a greater focus on quality education and the promotion of lifelong learning and development for all (UN, 2020). Shen et al. (2021) stated that students can develop higher-order thinking as well as competency-based literacy in STEAM activities. Furthermore, Ültay and Ültay (2020) stated that teacher education students are considered an important group for educational sustainability and that engaging in more STEAM activities can help them acquire 21st century skills such as collaboration and innovation. More importantly, related studies have also found that learners with higher learning performance in inquiry-based learning activities tend to have higher inquiry behaviors and continue to improve their scientific literacy in inquiry settings (Wen et al., 2020). In contrast, this study found that teacher education students’ STEAM learning performance was closely related to their sustainable inquiry behaviors, and teacher education students with higher STEAM learning performance tended to have higher sustainable inquiry behaviors and would be more willing to continue to engage in collaborative inquiry in future learning activities. Therefore, the findings of this study are consistent with previous research (Chen and Wang, 2020) in that if teacher education students have high learning performance when participating in STEAM activities, they are highly motivated to continue to engage in collaboration and inquiry learning.

## Conclusion and recommendations

### Conclusion

Within the SDG4 agenda, the implementation of quality education and lifelong learning for all is critical to achieving sustainable development in education, and the implementation of STEAM education in various learning environments not only supports the collaboration and development of learners, but also better enables sustainable development (Campbell et al., 2022). While more relevant studies have explored STEAM learning motivation and inquiry learning, little has been seen about the integration of short videos into STEAM education, and the application and effectiveness of short videos in STEAM education activities. The use of social media such as short videos will be beneficial for helping learners to promote their learning and development in various formal and informal settings.

Therefore, based on Sustainable Development Goal 4 (SDG4), this study used the triadic reciprocity theory and technology acceptance theory as its theoretical basis, a quasi-experimental research design. In this study of a 4-week quasi-experimental design combining STEAM educational activities with short videos, the research model was validated, and it was found that the higher the CS-E of teacher educators in STEAM education activities, the higher their PU of short videos and the higher their PEOU of short videos. Therefore, more use should be made of collaborative learning among teacher trainees to further enhance their ability and perception of applying short-form video learning by enhancing their sense of CS-E. In addition, higher CS-E of teacher education students can be enhanced by the application of short videos to STEAM education activities, they also had higher learning performance in STEAM and sustainable inquiry behaviors. Therefore, schools and teachers should provide more opportunities and resources for short video learning in STEAM education activities, and not only promote active learning among teacher education students, but also to enhance collaborative team learning by using social media such as short videos to guide teacher education students to participate more actively and proactively in STEAM activities and further enhance their STEAM learning performance and sustainable inquiry behaviors.

### Recommendations

In the development process of achieving SDG4, a sustainable development goal in education, more emphasis is being placed on developing 21st century skills such as inquiry and innovation in students (Partnership for 21st Century Skills, 2019; Singh, 2021). However, STEAM education activities in the past have focused more on fostering students’ creativity and have failed to promote students’ collaboration and inquiry learning based on STEAM activities. In addition, social media such as short videos have stronger visibility, interactivity, and richer resources, so adding more short video resources to STEAM education activities will help promote multi-directional interaction between students and short video resources, and so on, and apply short videos as educational resources in curriculum learning to better achieve sustainable education development. Therefore, in pre-service teacher training, teachers should integrate more short video resources into STEAM education activities and guide teacher trainees to use short video resources for collaborative learning in various formal and informal learning environments, to better achieve continuous learning and inquiry development.

In addition, short video resources and applications are beneficial for helping teacher education students better engage in collaborative learning and inquiry in STEAM activities, yet the learning performance in STEAM is low. Therefore, although short video platforms have a large number of video resources, because STEAM in preschool science education involves different scientific knowledge, inquiry methods, and artistic expression, teachers still need to be more targeted in guiding teacher trainees to choose appropriate short video resources and provide easy-to-understand and operate video resources to improve STEAM activity participation and further strengthen STEAM scientific knowledge and application in preschool education through various ways in order to enhance teacher trainees’ STEAM learning performance.

### Limitations and future study

According to the United Nations Sustainable Development Goal 4, more attention should be paid to the acquisition of lifelong learning skills such as information technology, arts, and vocational competencies in all types of courses (Lu et al., 2022), and more hands-on activities will help students engage in STEAM activities (Christensen et al., 2015). Although this study explored short video applications and STEAM collaborative learning and performance, it has not yet clarified how short videos affect teacher education students’ STEAM collaborative behaviors and outcomes. Therefore, the impact of short video applications on STEAM educational activities can be explored in subsequent studies based on the belief-action-outcome model.

In addition, Boice et al. (2021) indicated that qualitative research can provide a better understanding of learners’ perceptions of STEAM education, as well as insights into changes in their participation in STEAM activities. Although this study has confirmed the relationship between short video applications and teacher education students’ collaborative and inquiry learning, it was not possible to gain deeper insights into what kinds of methods and content of short videos have an impact on teacher education students’ STEAM collaborative learning. Therefore, a qualitative approach can be adopted in future research to understand teacher education students’ perceptions of short video applications and STEAM activities to further extend the findings. In addition, because this study focused on STEAM science learning among teacher education students in preschool education, the findings are limited to the subject of the preschool children’s science curriculum. However, the results of this study suggest that the effectiveness of short videos and STEAM activities needs to be further explored, and therefore, the effectiveness of the application of short videos and STEAM educational activities can be explored through different disciplinary contexts in subsequent studies.

Analysis of covariance (ANCOVA) is commonly used in social science research to help researchers better explore multiple variables and comparisons between interactions to better identify potential variables across groups (Leppink, 2018). Since this study only used basic statistical tools to explore the study variables and their relationships with each other, it failed to clearly and comprehensively identify the potential variables among the groups, so the analysis can be further extended in the follow-up study by using ANOCA to analyze the study variables and their effects on each other.

## Data availability statement

The raw data supporting the conclusions of this article will be made available by the authors, without undue reservation.

## Ethics statement

Ethical review and approval was not required for this study on human participants in accordance with the Local Legislation and Institutional Requirements. Written informed consent for participation was not required for this study in accordance with the National Legislation and the Institutional Requirements.

## Author contributions

All authors listed have made a substantial, direct, and intellectual contribution to the work, and approved it for publication.

## Funding

This study acknowledges the project funding for the vocational education teaching reform of Guangxi Education Department in 2021; the project name is “Research on Innovative Mode of Cultivating Talents in Preschool Education in Higher Education Institutions Based on “STEAM” Education Concept”; Grant number: GXGZJG2021B179.

## Conflict of interest

The authors declare that the research was conducted in the absence of any commercial or financial relationships that could be construed as a potential conflict of interest.

## Publisher’s note

All claims expressed in this article are solely those of the authors and do not necessarily represent those of their affiliated organizations, or those of the publisher, the editors and the reviewers. Any product that may be evaluated in this article, or claim that may be made by its manufacturer, is not guaranteed or endorsed by the publisher.
